# A new genus and two new species of Phygadeuontini (Hymenoptera, Ichneumonidae, Cryptinae) from China

**DOI:** 10.3897/zookeys.73.836

**Published:** 2010-12-29

**Authors:** Mao-Ling Sheng, Shu-Ping Sun

**Affiliations:** General Station of Forest Pest Management, State Forestry Administration, Shenyang, Liaoning, 110034, China

**Keywords:** Ichneumonidae, *Carinityla*, new genus, new species, taxonomy, China

## Abstract

Carinityla Sheng & Sun **gen. n.**, Carinityla punctulata Sheng & Sun **sp. n.** and Carinityla pilosa Sheng & Sun **sp. n.** belonging to the tribe Phygadeuontini of the subfamily Cryptinae (Hymenoptera, Ichneumonidae), collected from Jiangxi Province, China, are described. A key to the species of the new genus, Carinityla Sheng & Sun, **gen. n.**, is provided and the genus is placed in Townes’ key to genera of Endaseina.

## Introduction

According to the most recent catalogue of Ichneumonoidea (Yu et al. 2005), the tribe Phygadeuontini of the subfamily Cryptinae (Hymenoptera, Ichneumonidae), comprises 123 genera. Townes (1970) included 14 subtribes within Phygadeuontini (= Gelini of Townes). Two subtribes, Cephalobaridina and Gnypetomorphina, were subsequently synonymized with, respectively, the Phygadeuontina and Hemitelina by Horstmann (1992). Prior to this publication 33 genera and 76 species of Phygadeuontini have been recorded from China (Sheng and Sun 2009, Sheng and Zeng 2010, Sun and Sheng 2010).

In this article, one new genus and two new species, collected in Quannan County, Jiangxi Province, China, are described. The new genus belongs to the subtribe Endaseina of the tribe Phygadeuontini. Type specimens are deposited in the Insect Museum, General Station of Forest Pest Management, State Forestry Administration, People’s Republic of China.

The specimens were collected using the entomological net in the forest of Quannan County, Jiangxi Province (China). The forest of Quannan is a forest composed of mixed deciduous angiosperms and evergreen conifers, mainly including Quercus spp., Castania spp., Castanopsis fabri Hance, Cinnamomum spp., Pinus massoniana (Lamb.).

The morphological terminology is mostly that of Gauld (1991). Wing vein nomenclature is based on Ross (1936) and the terminology on Mason (1986, 1990).

## Taxonomy

### Carinityla

Sheng & Sun gen. n.

urn:lsid:zoobank.org:act:8C613E44-3B20-423D-B78A-DA983E05F4CF

#### Type species:

Carinityla punctulata Sheng & Sun, sp. n.

#### Etymology.

The name of the new genus is based on the strongly swollen tyloids. The gender is feminine.

#### Description.

**Figures 1–9.**
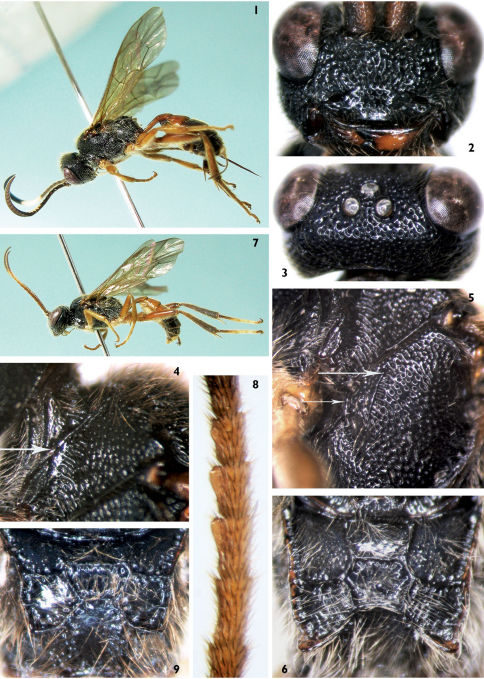
Carinityla punctulata Sheng & Sun, sp. n. **1–6**: Female. **1** Body, lateral view **2** Face **3** Vertex **4** Pronotum laterally **5** Mesopleuron **6** Propodeum. **7–9**: male. **7** Body, lateral view **8** Median portion of flagellomeres and tyloids **9** Propodeum.

**Figures 10–16.**
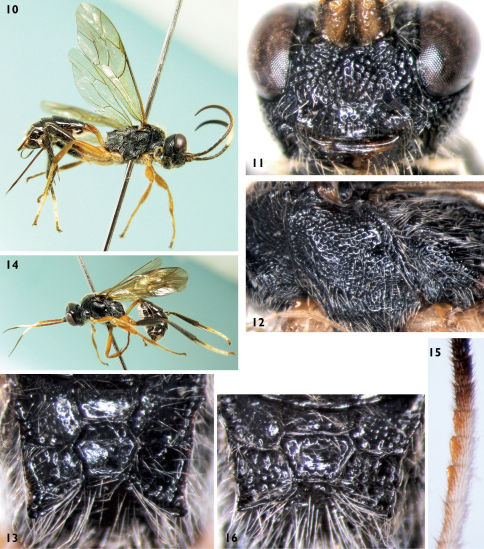
Carinityla pilosa Sheng & Sun, sp. n. **10–13**: Female. **10** Body, lateral view **11** Face **12** Mesopleuron **13** Propodeum **14–16**: male **14** Body, lateral view **15** Median portion of flagellomeres and tyloids **16** Propodeum.

Fore wing length 7.2 to 8.8 mm. Head and thorax with dense and comparatively long hairs. Eye surface with short, sparse hairs. Upper margin of face slightly produced, weakly concave medially. Clypeus slightly convex, median portion of apical margin somewhat arcuate and distinctly raised. Mandible elongate, upper and lower margins almost parallel, upper tooth longer than lower tooth. Apical truncation of scape almost transverse. Apical half of antenna strongly flattened below in female. Flagellomeres 10 to 11 (12) of male with strongly swollen tyloids. Epomia long and strong, from lower-anterior angle of pronotum continuing to its dorsal portion (Figure 4). Notauli present. Posterior edge of mesoscutum with transverse groove, which is unusually conspicuous and complete. Scutoscutellar groove without median longitudinal carina. Epicnemium with short transverse carina opposite lower corner of pronotum. Epicnemial carina strongly curved backward or broken (Figure 5) above sternaulus. Anterior half of sternaulus deep; posterior half weak, reaching to posterior margin of mesopleuron above its lower posterior corner. Areolet pentagonal, receiving vein 2m-cu at or slightly basad of its outer corner (Figures 1, 7, 10, 14). Vein 2m-cu subvertical, with one bulla. Hind wing vein 1-cu strongly inclivous, about 3.0 to 4.0 times as long as cu-a. Propodeum completely areolate, carinae very strong. Costula connecting area superomedia in front of its middle. Propodeal spiracle 3.0 to 3.5 times as long as wide. Median dorsal carinae of first tergum absent. Ovipositor compressed, tip very long and gradually tapered, with a weak nodus and very thin and indistinct ridges on ventral valve.

#### Remarks.

This new genus is similar to Amphibulus Kriechbaumer 1893 and Coptomystax Townes 1970 and can be distinguished from Amphibulus in the notaulus present; epicnemial carina strongly curved backward or secondary carina present above sternaulus; fore wing vein 2m-cu almost reaching 3rs-m (Figure 1, 7, 10, 14). In Amphibulus, the notaulus is absent; the epicnemial carina is neither curved backward nor is there a secondary carina above the sternaulus; fore wing vein 2m-cu is usually far from 3rs-m, usually connecting with the areolet near its median portion. The new genus can be distinguished from Coptomystax Townes by the eye surface with short sparse hairs; the upper margin of face without tubercle; a transverse groove at the posterior edge of mesoscutum distinct, complete and unusually conspicuous; the epicnemial carina approaching the subalar prominence, or discontinuous above the sternaulus. In Coptomystax the eye surface is bare; the upper edge of the face has a high and compressed median tubercle; the transverse groove at the posterior edge of the mesoscutum is distinct medially, evanescent laterally; the epicnemial carina ends below the middle of the hind edge of the pronotum.

This new genus can also be easily distinguished from the related genera Endasys Förster 1869 and Cisaris Townes 1970 by the following combination of characters: scutoscutellar groove without median longitudinal carina, median dorsal carinae of first tergum absent (Endasys: scutoscutellar groove with a median longitudinal carina, first tergum with distinct median dorsal carinae); fore wing with distinct areolet, posterior edge of mesoscutum with transverse groove (Cisaris: fore wing without areolet, posterior edge of mesoscutum without transverse groove).

In Townes’ (1970) key to genera, the new genus can be inserted as follows:

**Table:** 

7	Median dorsal carinae of first tergite strong, at least in front of spiracle. Prescutellar transverse groove with a strong median longitudinal carina. Holarctic Region	6. Endasys
–	Median dorsal carinae of first tergite weak or absent. Prescutellar transverse groove usually without a strong median longitudinal carina	7’
7’	Upper margin of face edge-shaped, without median tubercle. Apical half of antenna strongly flattened below in female. Areolet receiving vein 2m-cu at or slightly basad of its outer corner. Eye surface with sparse hairs	Carinityla Sheng & Sun, gen. n.
–	Upper margin of face concave, at least not edge-shaped, with a median tubercle. Apical half of antenna not flattened below. Areolet receiving vein 2m-cu near its center. Eye surface with or without hairs	8

#### Carinityla punctulata

Sheng & Sun sp. n.

urn:lsid:zoobank.org:act:8C613E44-3B20-423D-B78A-DA983E05F4CF

Figures 1–9 

##### Etymology.

The name of the new species is based on the dense punctures on the head and thorax.

##### Types.

*Holotype*, female, CHINA: Quannan County, 628m, Jiangxi Province, 9 June 2010, leg. Shi-Chang Li. *Paratypes*: 7 males, CHINA: Quannan County, 628 to 700m, Jiangxi Province, 16 May to 10 June 2008, leg. Shi-Chang Li. 1 female and 1 male, CHINA: Quannan County, 628 to 700m, Jiangxi Province, 26 to 31 May 2010, leg. shi-Chang Li.

##### Diagnosis.

Second tergum, hind femur and tibia reddish brown. Notaulus not reaching to center of mesoscutum. Scutellum weakly convex, lateral sides not raised, without carina except at its basal corner.

##### Description.

Female. Body length 9.3 to 9.7 mm. Fore wing length 7.2 to 7.8 mm. Ovipositor length about 2.8 mm. Head and mesosoma with dense punctures and long yellowish brown hairs.

##### Head.

Face (Figure 2) convex, approximately 2.2 times as wide as long, with dense, irregular punctures, median portion with short longitudinal wrinkles. Clypeal suture vestigial between clypeal foveae. Clypeus slightly convex, basal portion with punctures sparser than on face, subapically with shallow transverse concavity; apical portion smooth and impunctate, distinctly raised medially. Subbasal portion of mandible with short longitudinal wrinkles, apical portion with sparse shallow punctures; upper tooth distinctly longer than lower tooth. Cheek and gena with dense punctures, distance between punctures 0.2 to 0.5 times diameter of puncture. Subocular sulcus distinct. Malar space 0.4 to 0.5 times as long as basal width of mandible. Gena slightly convergent backward, in dorsal view 0.7 to 0.8 times as long as width of eye. Vertex (Figure 3) with dense punctures. Postero-ocellar line about 0.44 times as long as ocular-ocellar line. Frons approximately flat, with even and dense punctures, distance between punctures 0.2 to 0.5 times diameter of puncture. Antenna distinctly shorter than body length, with 27 flagellomeres, ratio of length of flagellomere 1:2:3:4:5 is 3.7:4.1:4.0:4.0:3.9. Flagellomeres 10 to 11 (12) of male with strongly swollen tyloids (Figure 8) on apical half of flagellomere and at most half as long as flagellomere. Occipital carina complete and strong, joining oral carina above base of mandible.

##### Mesosoma.

Anterior portion of pronotum with weak longitudinal wrinkles and fine punctures; laterally concave and lower portion with oblique transverse wrinkles; upper posterior portion with fine punctures, upper posterior margin shoulder-shaped, raised narrowly. Mesoscutum with dense elongate punctures. Notaulus present on front portion of mesoscutum. Scutoscutellar groove with very weak longitudinal wrinkles. Scutellum slightly convex, with irregular punctures. Postscutellum concave, smooth. Subalar prominence strongly convex. Anterior and upper margins of mesopleuron with fine punctures; median portion of mesopleuron (Figure 5) with irregular transverse punctures; speculum with dense and fine punctures. Epicnemial carina broken above sternaulus, upper end of lower portion connecting with short transverse carina opposite lower corner of pronotum; upper portion of epicnemial carina oblique, upper end reaching about half distance to subalar prominence. Metapleuron with dense punctures, distance between punctures 0.2 to 0.5 times diameter of puncture. Juxtacoxal carina complete. Anterior portion of submetapleural carina strongly lobed. Wings brownish hyaline. Fore wing with vein 1cu-a slightly distal of 1-M by less than vein width. Vein 2-Cu approximately 2.0 times as long as 2cu-a. Hind wing vein 1-cu about 3.0 times as long as cu-a. Legs robust, with dense brown hairs. Hind coxa and femur with distinct fine punctures. Spurs of hind tibia about half length of first tarsomere. Ratio of length of hind tarsomeres 1:2:3:4:5 is 10.0:4.5:3.5:1.8:3.7. Propodeum (Figure 6) with sandy beige long hairs. Area superomedia hexagonal, 1.2 times as wide as long, costula connecting slightly in front of its middle. Area basalis smooth, vaguely punctate. Area externa with distinct punctures. Area superomedia with indistinct longitudinal wrinkles. Area dentipara with oblique longitudinal wrinkles. Area lateralis with oblique transverse wrinkles. Area petiolaris with transverse wrinkles. Propodeal spiracle approximately 3.3 times as long as wide, almost touching lateral longitudinal carina (closer to lateral longitudinal carina than to pleural carina). Propodeal apophysis short and compressed.

##### Metasoma.

First and second terga smooth and shining, with very sparse and fine punctures. First tergum about 2.3 times as long as apical width. Postpetiole evenly convex. Median dorsal carinae absent. Dorsolateral and ventrolateral carinae complete. Spiracle circular, very small, slightly convex, located at apical 0.4 of first tergum. Second tergum 0.5 to 0.6 times as long as apical width. Remaining terga with short brown hairs. Ovipositor sheath approximately 0.95 times as long as hind tibia. Ovipositor compressed, with weak nodus.

##### Color

(Figure 1). Black, except the following. Ventral side and apical portion of scape, apical portion of pedicel, ventral side of basal portion (more or less) and flat side of flagellomeres, tegula brown. Dorsal sides of seventh to thirteenth flagellomeres white. Median portion of mandible, dorsal sides of front and mid femora and tibiae brown. Maxillary and labial palpi, fore and mid coxae, trochanters and ventral sides of femora yellowish brown. Fore and mid tarsi dark brown. Hind coxa brown to yellowish brown. Hind trochanter, femur and tibia reddish brown. Apical ends of hind femur and tibia, hind first tarsomere brownish black. Hind second to fifth tarsomeres blackish brown. First and second terga, basal margin of third tergum reddish brown. Posterior margin of third to sixth terga slightly narrowly tinged brown. Main portions of seventh and eighth terga white. Fore wing with stigma brown, veins blackish brown. Hind wing with veins brown.

##### Male

(Figure 7). Body length 9.5 to 11.0 mm. Fore wing length 7.2 to 8.5 mm. Face 1.7 to 1.8 times as wide as long. Antenna with 26 to 28 flagellomeres. Upper posterior portion of pronotum, in front of tegula, weakly convex. Notaulus present, almost reaching to center (about 0.4) of mesoscutum. Area superomedia inverse trapeziform, 1.9 to 2.1 times as wide as long, costula connecting at its anterior 0.2 (Figure 9). First tergum 2.6 to 2.7 times as long as apical width. Antennae with dorsal profiles of eighth to thirteen flagellomeres white. Apical half of hind first tarsomere and second to fourth tarsomeres buff. First to third terga reddish brown.

##### Host.

Unknown.

#### Carinityla pilosa

Sheng & Sun sp. n.

urn:lsid:zoobank.org:act:1F3772D3-2D07-45A3-985B-B547E618578C

Figures 10–16 

##### Etymology.

The specific name is derived from the long and dense hairs on the body.

##### Types.

*Holotype*, female, CHINA: Quannan County, 650m, Jiangxi Province, 29 June 2010, leg. Shi-Chang Li. *Paratypes*: 7 males, CHINA: Quannan County, 530 to 628m, Jiangxi Province, 12 May to 10 June 2008, leg. Shi-Chang Li. 3 males, CHINA: Quannan County, 628 to 700m, Jiangxi Province, 31 May to 18 June 2010, leg. Shi-Chang Li.

##### Diagnosis.

Second tergum, hind femur and tibia black. Notaulus of male reaching beyond center of mesoscutum. Lateral sides of scutellum raised and median portion weakly concave in male. Scutellum with lateral carina extending 0.2 to 0.3 of its length.

##### Description.

Female. Body length about 9.0 mm. Fore wing length about 7.2 mm. Ovipositor length about 2.8 mm. Head and mesosoma with long and dense yellowish brown hairs.

##### Head.

Face (Figure 11) strongly convex medially, approximately 1.9 times as wide as long, with dense and irregular punctures. Clypeal suture vestigial between clypeal foveae. Clypeus slightly convex, basal portion with sparse and irregular punctures, distance between punctures 1.0 to 3.0 times diameter of puncture; subapical portion with shallow, transverse concavity; apical 0.2 smooth and impunctate, median section of apical margin distinctly raised. Mandible with indistinct longitudinal wrinkles and fine punctures; upper tooth longer than lower tooth. Cheek with elongate punctures. Subocular sulcus indistinct. Malar space approximately 0.5 times as long as basal width of mandible. Gena with dense punctures, distance between punctures 0.2 to 1.0 times diameter of puncture; in dorsal view approximately 0.6 times as long as width of eye. Vertex with irregular punctures, distance between punctures 0.2 to 1.5 times diameter of puncture. Postero-ocellar line about 0.6 times as long as ocular-ocellar line. Frons approximately flat, with regular and dense punctures, distance between punctures 0.2 to 1.0 times diameter of puncture. Antenna distinctly shorter than body in length, with 27 flagellomeres, ratio of length of flagellomeres 1:2:3:4:5 is 3.7:4.7:4.5:4.3:4.1. Flagellomeres 10 to 11 (12) of male with tyloids (Figure 15) similar to those of Carinityla punctulata. Tyloid on flagellomere 11 0.7 to 0.9 times as long as flagellomere. Occipital carina complete and strong, joining oral carina above base of mandible.

##### Mesosoma.

Anterior portion of pronotum with fine punctures, laterally concave and lower portion with dense, oblique transverse wrinkles; upper posterior portion with fine punctures, distance between punctures 0.2 to 1.0 times diameter of puncture; upper posterior margin slightly and narrowly raised. Mesoscutum with dense punctures, distance between punctures 0.2 to 0.5 times diameter of puncture; posterior median portion with irregular longitudinal wrinkles. Notaulus present anteriorly. Scutoscutellar groove with fine longitudinal wrinkles. Scutellum almost flat, with irregular punctures. Postscutellum smooth and shining, lateral portion strongly convex, anterior-lateral portion deeply concave. Subalar prominence strongly convex. Upper portion of mesopleuron (Figure 12) with dense punctures; lower portion, above sternaulus, with irregular punctures; lower posterior portion with transverse wrinkles. Speculum with dense punctures. Epicnemial carina strongly curved backward above sternaulus, upper end reaching to subalar prominence. Metapleuron with dense and irregular punctures. Juxtacoxal carina complete. Anterior section of submetapleural carina strongly projecting. Wings brownish hyaline. Fore wing with vein 1cu-a almost opposite 1-M. Vein 2-Cu approximately 2.0 times as long as 2cu-a. Hind wing vein 1-cu about 3.0 times as long as cu-a. Legs robust, with long and dense brown hairs. Hind coxae and femora with distinct fine punctures. Spurs of hind tibia approximately half length of first tarsomere. Ratio of length of hind tarsomere 1:2:3:4:5 is 10.0:4.7:3.4:1.6:3.7. Propodeum (Figure 13) with long, brown hairs. Area superomedia hexagonal, approximately 1.15 times as wide as long, costula connecting slightly in front of its middle. Area basalis and area superomedia smooth and shining. Area externa with fine punctures. Area dentipara with indistinct wrinkles. Area spiracularis almost smooth. Area lateralis with oblique transverse wrinkles. Area petiolaris and area posteroexterna with transverse wrinkles. Propodeal apophysis short and compressed. Propodeal spiracle approximately 3.0 times as long as wide, distance to pleural carina approximately 1.3 times as long as distance to lateral longitudinal carina.

##### Metasoma.

First to third terga smooth and shining. First tergum approximately 2.3 times as long as apical width. Hind section of dorsolateral carina, behind spiracle, indistinct. Lateral margins of petiole almost parallel, only posterior portion slightly broadened. Postpetiole weakly and evenly convex, anterior half with sparse and fine punctures. Spiracle circular, very small, located at about apical 0.3 of first tergum. Second tergum approximately 0.65 times as long as apical width. Remaining terga with short brown hairs and indistinct punctures. Ovipositor sheath approximately as long as hind tibia. Ovipositor compressed, with weak nodus.

##### Color

(Figure 10). Black, except the following. Ventral profiles of scape and pedicel dark brown. Flat portion of flagellomeres more or less brown. Dorsal profiles of eighth to fourteenth flagellomeres white. Maxillary and labial palpi buff except dark bases. Median portion of mandible crimson. All coxae and trochanters, inner profiles of front and mid femora yellowish brown. Remaining portion of fore legs and mid femora brown. Apices of mid femora, mid tibiae and tarsi puce. Apical portion of first tarsomere of hind tarsi, second to fourth tarsomeres, posterior median portions of sixth and seventh terga, main portion of eighth tergum white. Petiole of first tergum yellowish brown; postpetiole reddish brown. Stigma yellowish brown. veins brownish black.

##### Male

(Figure 14). Body length 9.5 to 12.0 mm. Fore wing length 7.5 to 8.8 mm. Face 1.7 to 1.8 times as wide as long. Malar space 0.2 to 0.3 times as long as basal width of mandible. Antenna with 27 to 29 flagellomeres. Upper posterior portion of pronotum, in front of tegula, weakly convex. Notaulus long, reaching beyond center of mesoscutum. Lateral sides of scutellum raised, median portion weakly concave; basal 0.2 to 0.3 with lateral carina. Median portion of mesopleuron smooth and shining, impunctate. Area superomedia 1.5 to 1.6 times as wide as long, costula connecting at its anterior 0.3 (Figure 16). Propodeal spiracle 3.0 to 3.5 times as long as wide. First tergum 2.3 to 2.5 times as long as apical width. Dorsal profile of basal flagellomeres brownish black, ventral profile reddish brown; dorsal profiles of seventh to thirteen flagellomeres white; apical flagellomeres brownish black. Stigma and veins brownish black.

##### Host.

Unknown.

### Key to species of Carinityla Sheng & Sun

**Table:** 

1	Female	2
–	Male	3
2	Epicnemial carina complete, strongly curved backward above sternaulus. First tergum lacking hind portion of dorsolateral carina (behind spiracle). Hind femora and second tergum black	Carinityla pilosa Sheng & Sun, sp. n.
–	Epicnemial carina broken above sternaulus. Dorsolateral carina of first tergum complete. Hind femora and second tergum reddish brown	Carinityla punctulata Sheng & Sun, sp. n.
3	Notaulus reaching beyond center of mesoscutum. Lateral sides of scutellum raised, median portion weakly concave. Area superomedia of propodeum 1.5 to 1.6 times as wide as long. Hind femora and second to third terga black	Carinityla pilosa Sheng & Sun, sp. n.
–	Notaulus not reaching to center of mesoscutum. Scutellum normal, weakly convex, lateral sides slanting downwards. Area superomedia of propodeum 1.9 to 2.1 times as wide as long. Hind femora and second to third terga reddish brown	Carinityla punctulata Sheng & Sun, sp. n.

## Supplementary Material

XML Treatment for Carinityla
